# Biosynthesis of Quantum Dots and Their Therapeutic Applications in the Diagnosis and Treatment of Cancer and SARS-CoV-2

**DOI:** 10.34172/apb.2023.065

**Published:** 2022-12-06

**Authors:** Musa Moetasam Zorab, Navid Mohammadjani, Morahem Ashengroph, Mehran Alavi

**Affiliations:** ^1^Department of Physics, University of Halabja, Kurdistan Region, Iraq.; ^2^Department of Biological Science, Faculty of Science, University of Kurdistan, Sanandaj, Kurdistan, Iran.

**Keywords:** Biological synthesis, Microorganisms, Quantum dots, Cancer therapy, SARS-CoV-2

## Abstract

Quantum dots (QDs) are semiconductor materials that range from 2 nm to 10 nm. These nanomaterials (NMs) are smaller and have more unique properties compared to conventional nanoparticles (NPs). One of the unique properties of QDs is their special optoelectronic properties, making it possible to apply these NMs in bioimaging. Different size and shape QDs, which are used in various fields such as bioimaging, biosensing, cancer therapy, and drug delivery, have so far been produced by chemical methods. However, chemical synthesis provides expensive routes and causes *serious environmental* and health issues. Therefore, various biological systems such as bacteria, fungi, yeasts, algae, and plants are considered as potent eco-friendly green nanofactories for the biosynthesis of QDs, which are *both economic and environmentally* safe. The review aims to provide a descriptive overview of the *various microbial* agents for the *synthesis* of *QDs* and their biomedical applications for the diagnosis and treatment of cancer and SARS-CoV-2.

## Introduction

 Quantum dots (QDs) are semiconductor nanomaterials (NMs) with the size range 2-10 nm, which due to their tiny size, have different unique optoelectronic properties compared to their bulk.^1^ The green synthesis of nanoparticles (NPs) has been introduced as an alternative method to chemical synthesis as it is safe, nontoxic, and highly applicable for biomedical applications.^2-5^ Bio-based synthesis of QD is a type of bottom-up synthesis. Due to the proven applications of NMs, especially QDs in the field of biomedicine, and the need for developing cheaper and less polluting techniques, the biosynthesis of NP techniques, especially QDs, is developing gradually.^2,6^ NPs produced by biosynthesis are more stable and have a more controlled shape.^7^ The effects of the surface to volume ratio and quantum size on nanoscale cause them to exhibit new optical, electronic, magnetic, and structural properties and thus can be used in various fields of technology, especially in biomedicine.^8^ In this regard, finding new biological sources such as plants, fungi, and microbial strains with a higher ability to produce biocompatible NPs with various sizes and shapes is one of the important steps developing the application of NMs in biomedicine.^9-12^ Due to their special optical properties such as high brightness, photobleaching resistance, and highly good surface-to-volume ratio, QDs can be used in a variety of in-vitro and in-vivo bioimaging, the feature of which can be applied to enhance imaging techniques, especially in the diagnosis of the early stage of cancerous tumors.^13^ Further, because of the above-mentioned properties, various QDs have been introduced for treating microbial infections and cancer by assisting drug delivery mechanisms.^14-17^ As the main physical feature, QDs are extraordinary NMs owing to their tiny size generating physically confined electron cloud as the quantum confinement resulting in unique optical (emitting higher energy light in blue color) or electronic properties.^18^

 For semiconducting QDs, this property is resulted from the transition of an electron from the valence band to the conductance band, which the excited electrons can drop back into the valence band and release their energy as photoluminescence. In this way, optical applications of QDs are caused by their high extinction coefficient and optical nonlinearities suitable for all-optical systems.^19^ In the case of therapeutic applications, these NMs have shown anticancer property as well as antibacterial activity against various strains of Gram-negative and Gram-positive bacteria in a dose-dependent manner.^20^ However, the major disadvantages for biomedical application of QDs are potential toxicity in physiological conditions, poor aqueous stability and solubility, prone to photo-bleaching, complexity in controlling bio-distribution property for *in vivo* multiplex imaging.^13^

 Coronavirus disease 2019 (COVID-19), an infectious disease, caused by the severe acute respiratory syndrome coronavirus 2 (SARS-CoV-2) has been identified firstly in Wuhan, China in December 2019. Several antiviral drugs such as remdesivir/favipiravir (inactivator of RNA-dependent RNA polymerase) and lopinavir/ritonavir (the protease inhibitor) have been employed to hinder SARS-CoV-2.^21^ However, the major side effects of these drugs including liver dysfunction, chest tightness, dark-colored urine, flushing, headache, hives, itching, light-colored stools, nausea, vomiting, and thrombocytopenia have led to finding more effective micro and nano formulations.^22,23^ Antimicrobial activities of QDs have been reported by various studies.^15,24^ According to recent studies,^25-27^ NMs specifically QDs can be employed to diagnose and treat COVID-19. Nonetheless, the possibility of QD toxicity to cells in the human body is one of the main limitations in the development of their use, especially in the field of biomedicine.^28^

**Figure 1:**
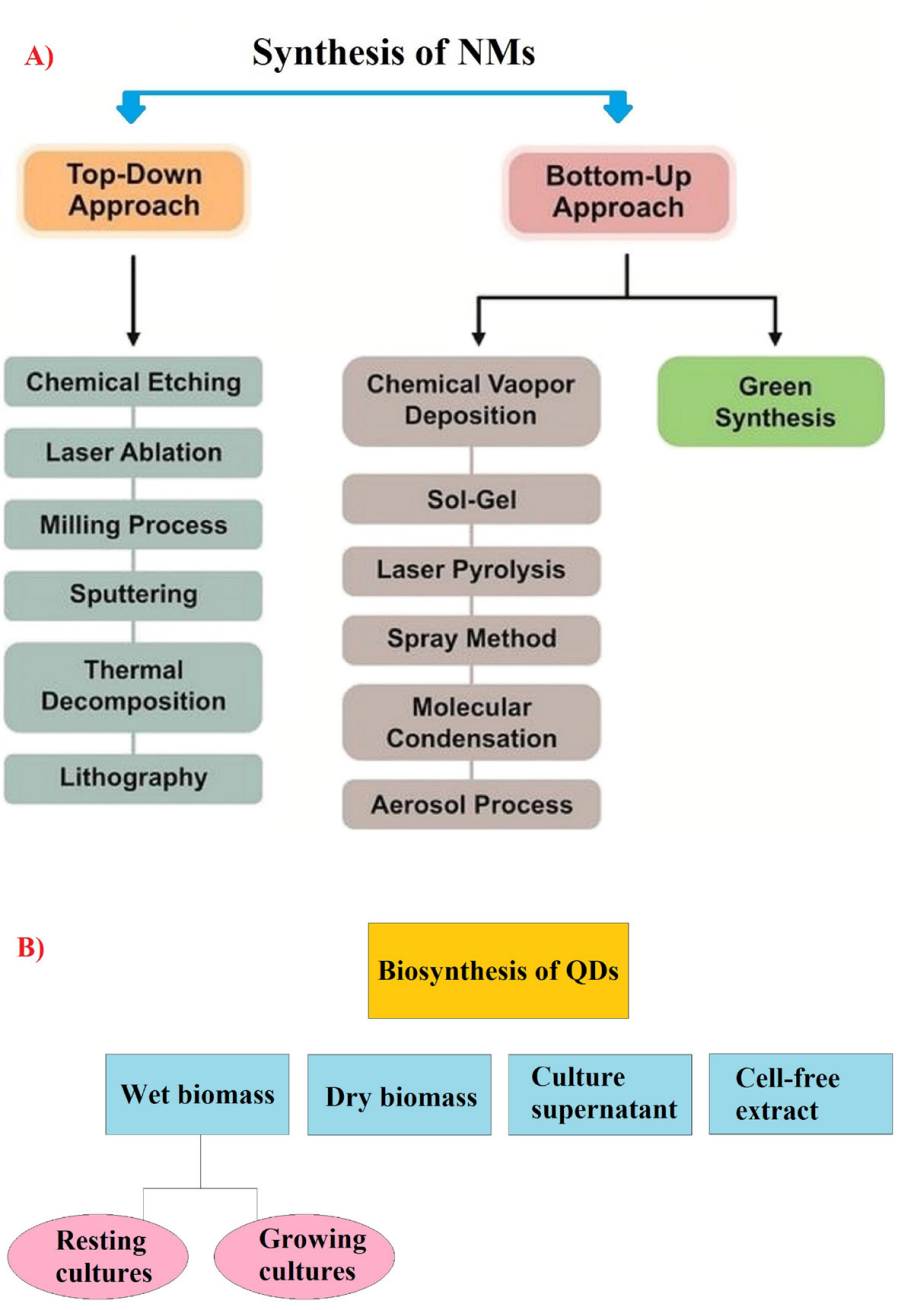
Two approaches for synthesis of NMs (A) and various strategies for biosynthesis of quantum dots (B). (*Note: *QD: Quantum dot). Copyright under the conditions of the Creative Commons Attribution (CC BY) license^43,44^

 Given the above-mentioned explanations, this study aimed to investigate and introduce QD biosynthesizing organisms for the development of green chemistry mechanisms in nanobiotechnology. There are top-down and bottom-up approaches as two main ways for synthesis of various NMs (Figure 1A).^29,30^ Top-down approach is majorly based on the physical methods such as X-ray lithography, molecular beam epitaxy, milling, and ion implantation, which high energy is needed to reduce size of bulk materials up to the nano scale (1-100 nm). In the case of bottom-up approach, in the colloidal solution, QDs are synthesized by self-assembly mechanism followed by chemical reduction.^11,31^ In fact, one of the negative points in many studies in the biomedical field is the use of chemical techniques for the development of QDs. As mentioned earlier, these chemical techniques are destructive to the environment. Nevertheless, no QD-based techniques and drugs have been observed in biomedical applications due to the toxicity and biocompatibility of the QDs synthesized with these chemical methods, as well as the high cost of synthesizing these QDs.^32^ In contrast, biosynthesis techniques can be considered as biocompatible and cost effective approach to prepare QDs.

## Biosynthesis of QDs

 To date, the biosynthesis of QDs has been reported in a variety of organisms.^33^ Among the various NP biosynthesis techniques, extracellular biosynthesis is more cost-effective than intracellular biosynthesis due to the lack of special treatments to purify the produced NPs.^2^ Preparation of NMs is the first step in nanobiotechnology.^29,34,35^ Biosynthetic organisms, when exposed to metal ions, accumulate them in or on their cell wall, which eventually leads to the production of NPs.^36^ It should be considered that the main goal is to obtain biocompatible QDs without disadvantages of expensive methods of laser irradiation, spray pyrolysis, electrolysis, and radiolysis or using toxic materials such as N,N-dimethylformamide, cetyl trimethylammonium bromide, and sodium dodecyl sulphate.^37^ There are three stages in the formation of NPs in living systems, including biodetoxification, biomineralization, and enzymatic reactions. Biodetoxification is the first organism response to toxic ions.^12,38,39^ Furthermore, biomineralization leads to the stabilization of NPs on the solid phase, and finally, enzymatic reactions lead to the growth of QDs.^33^ The main steps of each QD biosynthesis process are presented in the following sections.

## Biological synthesis of QDs under different strategies

 According to previous research, different strategies such as wet biomass (growing cultures/ resting cultures), culture supernatant, cell-free extract, and dry biomass are used in the biosynthesis process of different metal/metalloid NPs and QDs as intracellular or extracellular pathways (Figure 1A-B).^40-42^

## Evaluation of the effect of different parameters on QD synthesis

**Figure 2:**
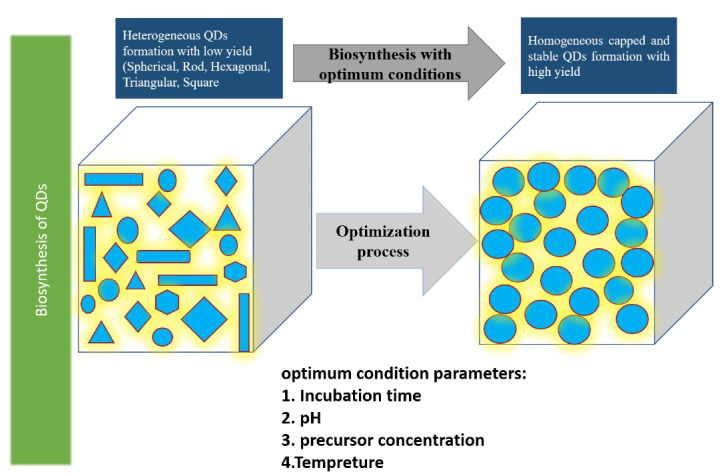
The effect of different parameters on the biosynthesis of QDs for detecting optimum conditions such as optimum pH, precursor concentration, temperature, and incubation time. Note: QD: Quantum dot^48^

 The effects of various parameters such as precursor concentration, temperature, pH, stirring time, reaction time, and inoculum amount can be evaluated on the yield, morphology, and size of the NPs. More precisely, the amount of produced QDs is majorly affected by the changing these parameters, thus this stage is highly important in the overall process of biosynthesis, the details of which are depicted in Figure 2.^33,45-47^

## Characterization techniques

 QDs have been characterized using different techniques, including UV-Vis spectroscopy, Fourier transform infrared spectroscopy, X-ray photoelectron spectroscopy, X-ray absorption spectroscopy, N_2_ adsorption-desorption, high-resolution transmission electron microscopy, scanning electron microscopy, energy dispersive X-ray analysis, dynamic light scattering, andX-ray diffraction.^28,49-51^

## QD biotoxicity tests

 Low toxicity includes requirements for the use of metal QDs in biomedical applications. The produced QDs must have the least amount of toxicity to vital factors in living cells. One of the advantages of QD biosynthesis is its safety due to the presence of capping proteins on the produced QD for reducing its toxicity. MTT assay and inhibition zone are the two most common tests at this stage. The bioassay of biological systems and the toxicity of produced QDs in-vitro are measured in MTT assay. In this technique, 3-2,5-diphenyltetrazolium bromide is used in 96 wells. Cell and sample uptake are then divided by control sample uptake and multiplied by 100 to determine cell viability. In the inhibition zone, model organisms such as *Escherichia coli* are applied, followed by employing the radius of the non-growth zone after a certain period of incubation to determine the toxicity of QDs.^28,52^ Table 1 summarizes a collection of different species used for the synthesis of QDs.

**Table 1:** Potential biological sources used for the biosynthesis of different QDs

**Type of QDs**	**Size and shape**	**Organism**	**Extracellular/Intracellular**	**Reference**	**Year**
CdSe	Spherical QDs with an average size of 11 nm	*Fusarium Oxysporum*	Extracellular	^53^	2007
CdTe	The size range of 2-3.6 nm with spherical shape	Yeast cells	Extracellular	^54^	2010
CdS	Spherical QDs by an average size of 6 nm	*Saccharomyces cerevisiae*	Extracellular	^55^	2012
CdTe	15–20 nm, spherical	*Fusarium oxysporum*	Extracellular	^51^	2013
CdS	A mean grain size of 2.56 nm with spherical shape	*Phanerochaete chrysosporium*	Extracellular	^56^	2014
CdS	A spherical shape and 4-5 nm in size	*Pleurotus ostreatus*	Extracellular	^57^	2015
CdSe	15 to 20 nm and spherical	*Saccharomyces cerevisiae*	Extracellular	^58^	2015
ZnS	4 nm and spherical	*Clostridiaceae*sp.	Extracellular	^40^	2016
CdS	6.11 nm and circular	*Fusarium oxysporum*f. sp.*lycopersici*	Extracellular	^59^	2017
ZnS	20–40 nm with a large agglomerated structure	SRB	Extracellular	^60^	2017
Ag	< 10nm and spherical	*Eichhornia crassipes*	Extracellular	^61^	2017
CdS	10 nm and spherical	*E. coli*	Extracellular	^47^	2017
CdS	< 20 nm and spherical	*Acidithiobacillus thiooxidans*	Extracellular	^62^	2018
CdS	Spherical shape with the size of 2.31, 2.59, and 2.59 nm for green, orange and yellow fluorescent QDs, respectively	*Pseudomonas fragi*	Extracellular	^63^	2019
CdSe	2 to 4 nm with cubic shape	*Providencia vermicola*	Extracellular	^46^	2019
ZnS	An average particle size of 6.5 nm with circular shape	SRB	Extracellular	^64^	2019
CdSe	An average size of 3.1 nm with spherical shape	*E. coli*	Extracellular	^65^	2019
CdS	Spherical morphology with a size of 2–7 nm	*Rhaphanus sativus* (hairy roots)	Extracellular	^66^	2020
CdS	A mean size of 6.7 nm with spherical shape	*Pseudomonas chlororaphis*	Extracellular	^45^	2020
CdSe	A narrow size distribution of 3.2 nm and spherical QDs	*Rhodotorula mucilaginosa*	Extracellular	^67^	2020
Ag_2_Se	Spherical QDs with an uniform size of 3.9 nm by	*Saccharomyces cerevisiae*	Extracellular	^49^	2021
ZnCdS	An average particle size of 6.12 nm in monodisperse spheres	SRB	Extracellular	^68^	2021
CdS	An average size of 6 nm with circular shape	*E. coli*	Intracellular	^69^	2011
CdTe	Spherical QDs with a mean diameter of 2.33 nm	*Lumbricus rubellus* (earth worm)	Intracellular	^70^	2013
CdS	Spherical shape by a diameter predominantly from 5 to 7 nm	Plant hairy root (*Linaria maroccana )*	Intracellular	^71^	2014
Ag_2_S	Spherical QDs and an average diameter of 5.21 nm	Wheat endosperm cells	Intracellular	^72^	2016
HgTe	Non-spherical shape with up to 20 nm in diameter	*Allium fistulosum*	Intracellular	^73^	2016
SnO_2_	A mean particle size of 7 nm and spherical morphology	*Clitoria ternatea*(plant)	Intracellular	^50^	2020
PbS	A particle size in the range 3.47–11.45 nm and spherical shape	*Pseudomonas aeruginosa*	Intracellular	^74^	2020
CdS	Sphere-shaped QDs with the size in the range 4.63-17.54 nm	*Pseudomonas aeruginosa*	Intracellular	^75^	2021

*Note*. CdS: Cadmium sulfide; ZnS: Zinc sulfide; Ag_2_Se: Silver selenide; SnO_2_: Tin(IV) oxide; CdTe: Cadmium telluride; CdSe: Cadmium Selenide; ZnCdS: Zinc cadmium sulfide; PbSe: Lead selenide; Ag_2_S: Silver sulfide; PbS: Lead sulfide; Ag: Silver; HgTe: Mercury telluride; SRB:Sulfate-reducing bacteria; *E. coli: Escherichia coli; *QD: Quantum dot.

## Diagnosis and treatment of cancer

 QDs, as semiconductor NMs, are most commonly used in the diagnosis, imaging, labeling, and treatment of various diseases. Highly applied QDs include CdS, CdTe, CdSe, PbSe, PbS, SnTe, PbTe, lnP, and lnAs, but Cd-based QDs have the most applications due to their unique physicochemical properties.^76^ The unique photoluminescence properties of QDs have led to their application in in-vitro and in-vivo imaging, and they can also play an important role in drug delivery processes used for cancer treatment. Due to the toxicity of ordinary QDs, carbon dots have been considered for radiotherapy applications.^77^ According to some findings, carbon dots can damage bacteria, yeasts, and other organisms by generating oxygen radicals, but it is not yet clear whether these substances can damage human cells.^1^ This antibacterial mechanism is critical issue to hindering pathogenic bacteria specifically antibiotic-resistance ones.^78,79^ However, the use of QDs in bioimaging is limited due to their low specificity and inability to detect early-stage cancer tumors.^80^ Produced QDs by biological sources make these QDs biocompatible in bioimaging and anti-cancer drug applications. In fact, different metals may normally be non-toxic in the body but become toxic when their structure is at the quantum level. Therefore, determining the biotoxicity of the applied QDs is highly important in preclinical studies in this field.^81^

## Bioimaging of cancer cells

**Figure 3:**
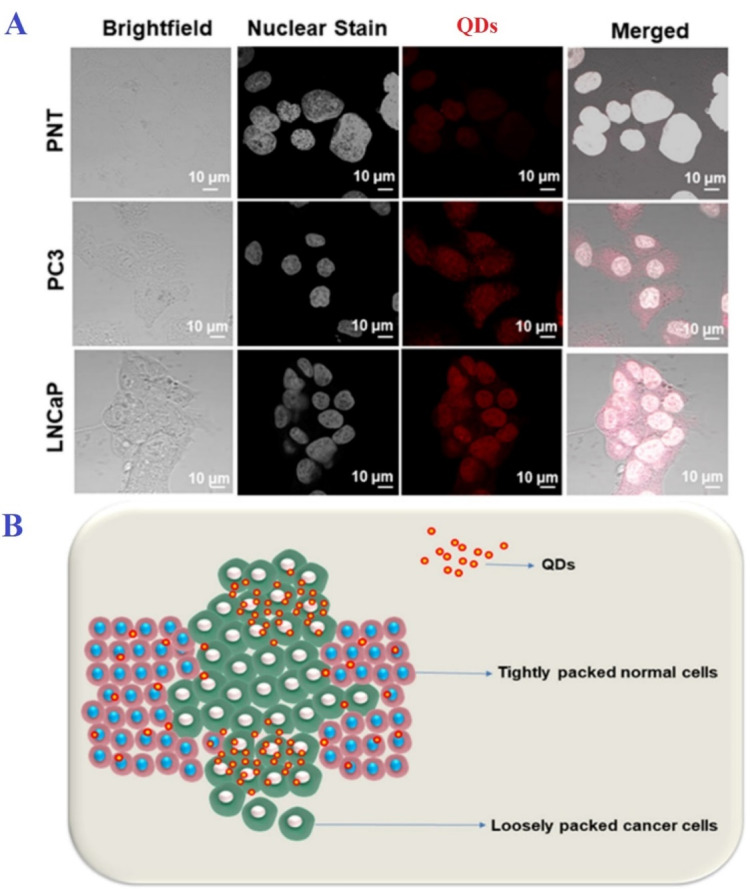
A) *In vitro* bioimaging of prostate cancer cells (PC3), normal prostate cells (PNT), and human adenocarcinoma cells (LNCaP); B) Possible pathway for the uptake of QDs in normal and cancer cells. *Note*. QD: Quantum dot. As shown, AgInSe/ZnS QDs could distinguish cancer cells from non-cancerous cells. Copyright under the conditions of the Creative Commons Attribution (CC BY) license^85^

 Detection of cancer in the early stages can reduce its risks by 30-50%. Therefore, due to their extraordinary fluorescent properties, QDs can be used in bioimaging to identify cancer cells, even in extremely small tumors.^82^ Conventional imaging techniques such as MRI and photoluminescence are unable to accurately detect cancerous tumors, especially in small cases due to their low resolution, and have a significant error rate. Accordingly, QDs have been proposed to increase the resolution of these imaging techniques.^83^ In other words, bioimaging by QDs is based on the identification of the specific biomarkers of cancer cells and is divided into in-vitro and in-vivo categories. In in-vitro bioimaging, numerous studies have been considered to develop the use of QDs to image the cancer cells of melanoma, ovary, breast, pancreatic, glioblastoma, ovarian epidermoid, lung, hepatocellular, and adenocarcinoma.^1,84^ For example, in a study by Ncapayi et al., prostate cancer cells with high specificity, compared to normal prostate cells, were imaged by AgInSe/ZnS QDs (Figure 3).^85^ In in-vivo bioimaging, the biomarkers of cancerous tumors by binding to QDs are involved in in-vivo imaging techniques.^1^ In these techniques, fluorescent QDs are injected into mice, and their fluorescent activity is identified by detectors.^86^

 The stability of the applied QD during the imaging process is one of the important points in bioimaging. Studies demonstrate that the biological samples are exposed to QDs after a while, and their illumination represents a significant reduction. Thus, Kim et al. used a CdSeZnS/ZnS QD alloy alongside multi-layered QDs. Finally, the QD-Cd alloy produced extremely sharper images. This sharpness was more evident in both in-vivo and in-vitro bioimaging.^87^ In addition, Ag–In–S/ZnS (AIS/ZnS) QDs with fluorescent emission in red color were used to *In vivo* fast imaging of the rat lymphatic tumor.^88^

## Cancer drug targeting

**Figure 4:**
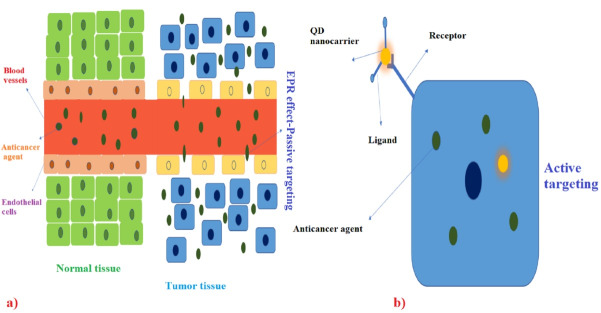
Passive and active targeting in cancer therapy by QDs nanocarriers. Note: QD: quantum dot^94^

 Chemotherapy is one of the main methods of killing cancer cells because cancer cells have a higher growth rate compared to normal cells. However, chemotherapy targets cells with a high growth rate, some normal cells (Skin, hair follicles, and the cells of the gastrointestinal tract) also have high growth rates and are affected by this treatment. Nanocarriers can target cancer cells efficiently by passive and active targeting as two main targeting methods.^89^ Passive targeting is based on the property of the enhanced permeability effect, which occurs due to the permeability of the arteries in the tumor-containing area and causes anti-cancer drugs to spread more in the tumor-containing areas.^90^ In active targeting, the specific and altered surface of cancer cells and their ability to bind to anti-cancer drugs are specifically applied in this technique (Figure 4).^91^ Nanocarriers increase the half-life of the drug in the body, increase the solubility of anti-tumor substances, and can play a highly significant role in enhancing the performance of anti-cancer drugs.^92,93^ The structure of QD-based carriers includes a core, a shell, and a capping structure on them. Graphene QDs, datum Cd QDs, and carbon QDs are typically used as the major carriers of anticancer drugs. The discussion of the difference in the function of cancerous tumors from one person to another is one of the main challenges in the development of the anti-cancer quantum drug carrier, and this personalization is extensively important in therapeutic discussions, as well as the unknown factors in different cancers. This is the next challenge that must be solved in the future. Highly sensitive DNA nanostructures with a hybrid structure can be extremely important candidates in the field of drug delivery.^94^ Some studies have combined QDs applied in biomedical fields, including carbon and Cd-based QDs, with magnetic NPs to use both specific optical and magnetic properties in combination in drug delivery processes in order to enhance the performance of nanocarriers. It is noteworthy that the biotoxicity of these manufactured nanocarriers is one of the main limitations in this field.^95^ The mentioned nanocarriers cannot be employed for metastatic tumors. Another discussion is the development of more cost-effective nanocarriers in this field that may enable the development of effective and cost-effective anti-cancer nanocarriers in the future.^96^ QDs can bind to antibodies, peptides, small molecules, and the like and deliver substances with medicinal properties to their target cells with high specificity. Various anti-cancer drugs such as doxorubicin (ZnO QDs), cisplatin (graphene QDs), and paclitaxel (ZnSe:Mn/ZnS QDs) have been linked to different QDs, and research is ongoing to find the best QDs to connect the identified common anticancer drugs.^97^ As shown in Figure 4, QDs nanocarriers can be used for passive and active targeting in cancer therapy.^94^

## Application of QDs in detecting and treatment of COVID-19

**Figure 5:**
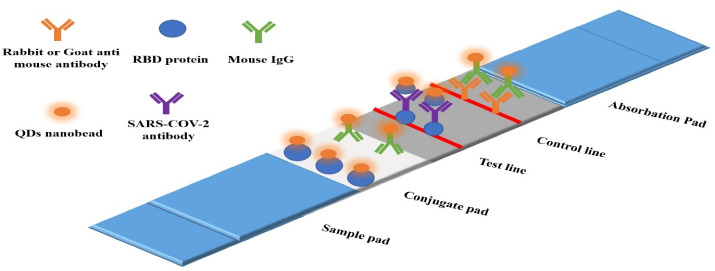
Overview of SARS- CoV-2 detective kit based on QDs nanobeads. *Note*. SARS-CoV-2: Severe acute respiratory syndrome coronavirus 2; RBD: Receptor binding domain; QDs: Quantum dots; IgG: Immunoglobulin G^103,105^

 The virus-sensitive properties particularly electrochemical and biochemical features for the production of next-generation viral biosensors have been utilized for detecting various pathogens.^98,99^ In this technique, the specific antibody of each virus is placed on the probe in contact with a QD, and the virus-related antigen can be detected by various detectors such as spectroscopy and the like if it is attached to the antibody on the fluorescent prop.^25,100^ The production of strong and detectable light with an extremely small amount of QDs is the main advantage of using QDs in the preparation of virus detector props over fluorescent proteins, representing the extensively high sensitivity of QDs in detecting viruses.^101^ The COVID-19 pandemic has killed approximately 6 million people by March 2022 around the world.^102^ The development of new and highly sensitive and cost-effective techniques in the field of SARS-CoV-2 virus detection is one of the main steps, along with extensive and comprehensive vaccination in the field of pandemic control. In the use of the QD props for SARS-CoV-2 detection, props are based on the detection of antibodies, antigens, and RNA/DNA (Table 2). In this regard, Li et al. developed a kit based on the lateral flow assay technique that detects antibodies to the virus with high specificity.^103^ One of the key benefits of using QDs is the lack of a need for initial pretreatment for the real-time polymerase chain reaction, minimizing the risk of aerosols and thus laboratory personnel (Figure 5).^104^

**Table 2:** Different QDs reported for SARS-CoV-2 detection

**Type of QDs**	**Description**	**Reference**
Polystyrene-based QDs	Lateral flow assay for identifying the SARS-CoV-2 virus by antibody detection	^103^
Magnetic graphene GQDs	Detection of SARS-CoV-2 by ultra-low field NMR relaxometry with low price (1.25 USD)	^104^
PbS Colloidal QDs	Electronic labeling strategy of protein has advantages over the standard ELISA technique	^106^
Niobium carbide MXene QDs	Use for identifying the N-gene of SARS-CoV-2	^107^
SQS QDs	Representation of 100% sensitivity in modified QDs compared to RT-PCR technique	^108^
CdTe QDs	Detection of RNA or DNA from SARS-CoV-2 using FRET experiment	^109^

*Note*. SARS-CoV-2: Severe acute respiratory syndrome coronavirus 2; QD: Quantum dots; RT-PCR: Real-time polymerase chain reaction; NMR: Nuclear magnetic resonance; ELISA: Enzyme-linked immunosorbent assay; FRET: Fluorescence resonance energy transfer.

## COVID-19 treatment

**Figure 6:**
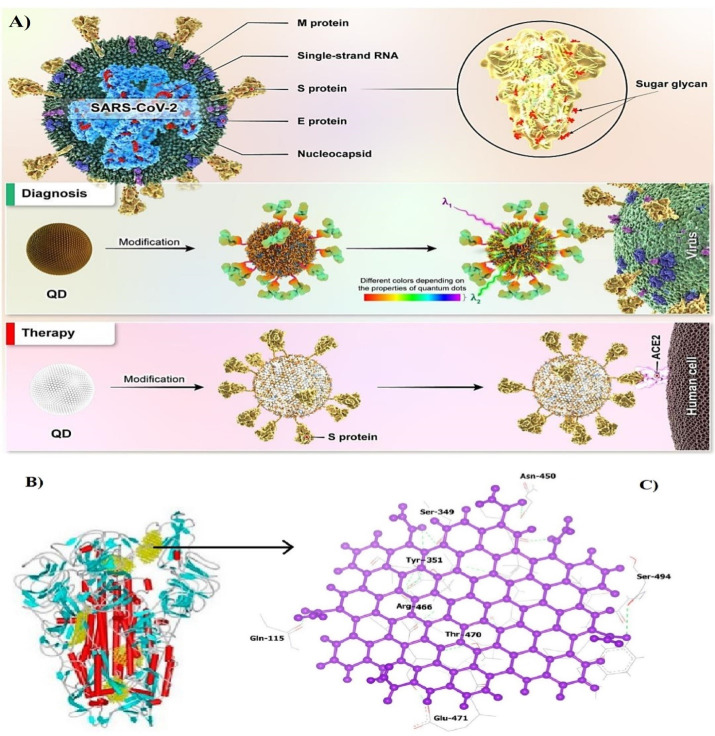
A) The inactivation and analysis of SARS-CoV-2 by QDs. B) Molecular docking of NH_2_-OH-CQD molecules within SARS-CoV-2 S protein, C) overview of the connection of NH2-OH carbon QDs to RBD. *Note*. NH2-OH: Hydroxylamine; QD: Quantum dots; RBD: Receptor binding domain^26,105^

 SARS-CoV-2 binds to angiotensin-converting enzyme 2 (ACE2) receptors through its receptor-binding domain (RBD) portion. In a bioinformatics study by Ramezani et al, connectivity between carbon QDs binding energy levels (-699.3 kJ/mole) have been extremely lower than favipiravir as effective nonspecific antiviral drugs (-487.2 kJ/mole), indicating the potential of carbon QD as an antivirus that can be prescribed at a lower dose and with fewer side effects for treating COVID-19 patients. The main function of these QDs is to cover the RBD to prevent the virus from attaching to the ACE2 receptor (Figure 6).^26^ Additionally, QDs can act as a delivery system for COVID-19 vaccines and drugs to reduce the side effects of various COVID-19 drugs with high accuracy by taking lower doses of drugs. According to some studies, QDs can also be useful in the development of inhibitory drugs.^110^

## Conclusion

 This review article attempted to explain the general principles of QD biosynthesis and biomedical applications as an effective, environmentally-friendly, biocompatible, and cost-effective technique. To develop this green technique, we must continue to seek new organisms in nature. There must be potentially undiscovered biosynthetic strains in nature that have not been tested yet. The application of QDs has been proven in the diagnosis and treatment of different illnesses. This review focused on investigating the use of QDs for diagnosing and treating two important and deadly diseases of the present age, namely, COVID-19 and cancer.

## Competing Interests

 Authors declare no conflict of interests.

## Ethical Approval

 Not applicable.

## References

[1] Devi S, Kumar M, Tiwari A, Tiwari V, Kaushik D, Verma R (2022). Quantum dots: an emerging approach for cancer therapy. Front Mater.

[2] Soosani N, Ashengroph M, Chehri K (2021). Extracellular green synthesis of zinc oxide nanoparticle by using the cell-free extract Rhodotorulapacifica NS02 and investigation of their antimicrobial activities. Nova BiologicaReperta.

[3] Borovaya MN, Burlaka OM, Yemets AI, Blume YB. Biosynthesis of quantum dots and their potential applications in biology and biomedicine. In: Fesenko O, Yatsenko L, eds. Nanoplasmonics, Nano-Optics, Nanocomposites, and Surface Studies. Cham: Springer; 2015. p. 339-62. 10.1007/978-3-319-18543-9_24.

[4] Ashengroph M. Isolation and characterization of a native strain of Aspergillus niger ZRS14 with capability of high resistance to zinc and its supernatant application towards extracellular synthesis of zinc oxide nanoparticles. Biological Journal of Microorganism 2013;2(7):29-44. [Persian].

[5] Ashengroph M, Hosseini SR (2019). Synthesis analysis and antibacterial activity of selenium nanoparticles produced by Pseudomonas alcaligenes. J Microbial World.

[6] Alavi M, Rai M, Martinez F, Kahrizi D, Khan H, Rose Alencar de Menezes I (2022). The efficiency of metal, metal oxide, and metalloid nanoparticles against cancer cells and bacterial pathogens: different mechanisms of action. Cell Mol Biomed Rep.

[7] Mohammadi Bolbanabad E, Ashengroph M, Darvishi F (2020). Development and evaluation of different strategies for the clean synthesis of silver nanoparticles using Yarrowialipolytica and their antibacterial activity. Process Biochem.

[8] Ashengroph M, Khaledi A (2018). Rapid extracellular synthesis of cadmium sulfide nanoparticles by Pseudomonas pseudoalcaligenes Cd11 and study of its antibacterial activity. Cell Mol Res.

[9] Ashengroph M, Hosseini SR (2021). A newly isolated Bacillus amyloliquefaciens SRB04 for the synthesis of selenium nanoparticles with potential antibacterial properties. Int Microbiol.

[10] Alavi M, Rai M (2021). Antisense RNA, the modified CRISPR-Cas9, and metal/metal oxide nanoparticles to inactivate pathogenic bacteria. Cell Mol Biomed Rep.

[11] Alavi M, Webster TJ, Li L (2022). Theranostic safe quantum dots for anticancer and bioimaging applications. Micro Nano Bio Asp.

[12] Alavi M, Rai M, Rose Alencar de Menezes I (2022). Therapeutic applications of lactic acid bacteria based on the nano and micro biosystems. Nano Micro Bios.

[13] Wagner AM, Knipe JM, Orive G, Peppas NA (2019). Quantum dots in biomedical applications. Acta Biomater.

[14] Molaei MJ (2019). Carbon quantum dots and their biomedical and therapeutic applications: a review. RSC Adv.

[15] Alavi M, Jabari E, Jabbari E (2021). Functionalized carbon-based nanomaterials and quantum dots with antibacterial activity: a review. Expert Rev Anti Infect Ther.

[16] Behzadmehr R, Rezaie-Keikhaie K (2022). Evaluation of active pulmonary tuberculosis among women with diabetes. Cell Mol Biomed Rep.

[17] Muhammad I, Sale PM, Salisu MK, Muhammad TM, Abubakar B, Maidala AL (2022). Molecular analysis of bio-makers of chloroquine resistance in Plasmodium falciparum isolate from Gombe local government area, Gombe state, Nigeria. Cell Mol Biomed Rep.

[18] Mohamed WA, Abd El-Gawad H, Mekkey S, Galal H, Handal H, Mousa H (2021). Quantum dots synthetization and future prospect applications. Nanotechnol Rev.

[19] Singh S, Raina D, Rishipathak D, Babu KR, Khurana R, Gupta Y (2022). Quantum dots in the biomedical world: a smart advanced nanocarrier for multiple venues application. Arch Pharm (Weinheim).

[20] Ali OM, Hasanin MS, Suleiman WB, Helal EE, Hashem AH. Green biosynthesis of titanium dioxide quantum dots using watermelon peel waste: antimicrobial, antioxidant, and anticancer activities. Biomass Convers Biorefin 2022. 10.1007/s13399-022-02772-y.

[21] Jean SS, Lee PI, Hsueh PR (2020). Treatment options for COVID-19: the reality and challenges. J Microbiol Immunol Infect.

[22] Alavi M, Asare-Addo K, Nokhodchi A (2020). Lectin protein as a promising component to functionalize micelles, liposomes and lipid NPs against coronavirus. Biomedicines.

[23] Öztürk Ergür F, Yıldız M, Şener MU, Kavurgacı S, Ozturk A (2022). Adverse effects associated with favipiravir in patients with COVID-19 pneumonia: a retrospective study. Sao Paulo Med J.

[24] Yan C, Wang C, Hou T, Guan P, Qiao Y, Guo L (2021). Lasting tracking and rapid discrimination of live gram-positive bacteria by peptidoglycan-targeting carbon quantum dots. ACS Appl Mater Interfaces.

[25] Nasrin F, Chowdhury AD, Takemura K, Park EY (2020). Fluorometric sensing platform based on localized surface plasmon resonance using quantum dots-gold nanocomposites optimizing the linker length variation. Biophys J.

[26] Ramezani Z, Dayer MR, Noorizadeh S, Thompson M (2021). Deactivation of SARS-CoV-2 via shielding of spike glycoprotein using carbon quantum dots: bioinformatic perspective. COVID.

[27] Rahbar-Karbasdehi E, Rahbar-Karbasdehi F (2021). Clinical challenges of stress cardiomyopathy during coronavirus 2019 epidemic. Cell Mol Biomed Rep.

[28] Qi S, Chen J, Bai X, Miao Y, Yang S, Qian C (2021). Quick extracellular biosynthesis of low-cadmium ZnxCd1−xS quantum dots with full-visible-region tuneable high fluorescence and its application potential assessment in cell imaging. RSC Adv.

[29] Alavi M, Rai M, Varma RS, Hamidi M, Mozafari MR (2022). Conventional and novel methods for the preparation of micro and nanoliposomes. Micro Nano Bio Asp.

[30] Alavi M, Mozafari MR, Hamblin MR, Hamidi M, Hajimolaali M, Katouzian I (2022). Industrial-scale methods for the manufacture of liposomes and nanoliposomes: pharmaceutical, cosmetic, and nutraceutical aspects. Micro Nano Bio Asp.

[31] Valizadeh A, Mikaeili H, Samiei M, Mussa Farkhani S, Zarghami N, Kouhi M (2012). Quantum dots: synthesis, bioapplications, and toxicity. Nanoscale Res Lett.

[32] Desmond LJ, Phan AN, Gentile P (2021). Critical overview on the green synthesis of carbon quantum dots and their application for cancer therapy. Environ Sci Nano.

[33] Zhou J, Yang Y, Zhang CY (2015). Toward biocompatible semiconductor quantum dots: from biosynthesis and bioconjugation to biomedical application. Chem Rev.

[34] Ashengroph M (2014). Extracellular synthesis of silver nanoparticles by Ralstonia sp. SM8 isolated from the Sarcheshmeh copper mine. Biological Journal of Microorganism.

[35] Amraei S, Ahmadi S (2022). Recent studies on antimicrobial and anticancer activities of saponins: a mini-review. Nano Micro Bios.

[36] Ashengroph M, Sahami-Soltani M (2018). Antimicrobial effects of extracellular copper sulfide nanoparticles synthesized from Bacillus licheniformis. J Microbial World.

[37] Sasidharan S, Poojari R, Bahadur D, Srivastava R (2018). Embelin-mediated green synthesis of quasi-spherical and star-shaped plasmonic nanostructures for antibacterial activity, photothermal therapy, and computed tomographic imaging. ACS Sustain Chem Eng.

[38] Alavi M, Hamblin MR, Martinez F, Aghaie E, Khan H, Rose Alencar de Menezes I (2022). Micro and nanoformulations of insulin: new approaches. Nano Micro Bios.

[39] Barik A, Biswal D, Arun A, Balasubramanian V. Biodetoxification of heavy metals using biofilm bacteria. In: Mishra BB, Nayak SK, Mohapatra S, Samantaray D, eds. Environmental and Agricultural Microbiology. Wiley; 2021. p. 39-61.

[40] Yue L, Qi S, Wang J, Cai J, Xin B (2016). Controllable biosynthesis and characterization of α-ZnS and β-ZnS quantum dots: comparing their optical properties. Mater Sci Semicond Process.

[41] Alavi M, Thomas S, Sreedharan M (2022). Modification of silica nanoparticles for antibacterial activities: mechanism of action. Micro Nano Bio Asp.

[42] Alavi M, Hamblin MR, Martinez F, Kennedy JF, Khan H (2022). Synergistic combinations of metal, metal oxide, or metalloid nanoparticles plus antibiotics against resistant and non-resistant bacteria. Micro Nano Bio Asp.

[43] Jeyaraj M, Gurunathan S, Qasim M, Kang MH, Kim JH (2019). A comprehensive review on the synthesis, characterization, and biomedical application of platinum nanoparticles. Nanomaterials (Basel).

[44] Alavi M, Hamblin MR, Kennedy JF (2022). Antimicrobial applications of lichens: secondary metabolites and green synthesis of silver nanoparticles: a review. Nano Micro Biosy.

[45] Ashengroph M, Khaledi A, Bolbanabad EM (2020). Extracellular biosynthesis of cadmium sulphide quantum dot using cell-free extract of Pseudomonas chlororaphis CHR05 and its antibacterial activity. Process Biochem.

[46] Abou-Assy RS, El-Deeb BA, Al-Talhi AD, Mostafa NY (2019). Biosynthesis of cadmium selenide quantum dots by Providencia vermicola. Afr J Microbiol Res.

[47] Yan ZY, Du QQ, Qian J, Wan DY, Wu SM (2017). Eco-friendly intracellular biosynthesis of CdS quantum dots without changing Escherichia coli’s antibiotic resistance. Enzyme Microb Technol.

[48] Han XL, Li Q, Hao H, Liu C, Li R, Yu F (2020). Facile one-step synthesis of quaternary AgInZnS quantum dots and their applications for causing bioeffects and detecting Cu2. RSC Adv.

[49] Liu J, Zheng D, Zhong L, Gong A, Wu S, Xie Z (2021). Biosynthesis of biocompatibility Ag2Se quantum dots in Saccharomyces cerevisiae and its application. BiochemBiophys Res Commun.

[50] Fatimah I, Sahroni I, Muraza O, Doong R-a (2020). One-pot biosynthesis of SnO2 quantum dots mediated by Clitoriaternatea flower extract for photocatalytic degradation of rhodamine B. J Environ Chem Eng.

[51] Syed A, Ahmad A (2013). Extracellular biosynthesis of CdTe quantum dots by the fungus Fusarium oxysporum and their anti-bacterial activity. Spectrochim Acta A Mol BiomolSpectrosc.

[52] Yan Z, Qian J, Gu Y, Su Y, Ai X, Wu S (2014). Green biosynthesis of biocompatible CdSe quantum dots in living Escherichia coli cells. Mater Res Express.

[53] Kumar SA, Ansary AA, Ahmad A, Khan MI (2007). Extracellular biosynthesis of CdSe quantum dots by the fungus, Fusarium oxysporum. J Biomed Nanotechnol.

[54] Bao H, Hao N, Yang Y, Zhao D (2010). Biosynthesis of biocompatible cadmium telluride quantum dots using yeast cells. Nano Res.

[55] Huang HQ, He MX, Wang WX, Liu JL, Mi CC, Xu SK. [Biosynthesis of CdS quantum dots in Saccharomyces cerevisiae and spectroscopic characterization]. Guang Pu Xue Yu Guang Pu Fen Xi 2012;32(4):1090-3. [Chinese]. 22715791

[56] Chen G, Yi B, Zeng G, Niu Q, Yan M, Chen A (2014). Facile green extracellular biosynthesis of CdS quantum dots by white rot fungus Phanerochaetechrysosporium. Colloids Surf B Biointerfaces.

[57] Borovaya M, Pirko Y, Krupodorova T, Naumenko A, Blume Y, Yemets A (2015). Biosynthesis of cadmium sulphide quantum dots by using Pleurotusostreatus (Jacq.) P. Kumm. BiotechnolBiotechnol Equip.

[58] Wu SM, Su Y, Liang RR, Ai XX, Qian J, Wang C (2015). Crucial factors in biosynthesis of fluorescent CdSe quantum dots in Saccharomyces cerevisiae. RSC Adv.

[59] Sandoval-Cárdenas I, Gómez-Ramírez M, Rojas-Avelizapa NG (2017). Use of a sulfur waste for biosynthesis of cadmium sulfide quantum dots with Fusarium oxysporum f. sp. lycopersici. Mater Sci Semicond Process.

[60] Murray AJ, Roussel J, Rolley J, Woodhall F, Mikheenko IP, Johnson DB (2017). Biosynthesis of zinc sulfide quantum dots using waste off-gas from a metal bioremediation process. RSC Adv.

[61] Silva A, Martinez-Gallegos S, Rosano-Ortega G, Schabes-Retchkiman P, Vega-Lebrun C, Albiter V (2017). Nanotoxicity for E. coli and characterization of silver quantum dots produced by biosynthesis with Eichhornia crassipes. J Nanostruct.

[62] Ulloa G, Quezada CP, Araneda M, Escobar B, Fuentes E, Álvarez SA (2018). phosphate favors the biosynthesis of CdS quantum dots in Acidithiobacillusthiooxidans ATCC 19703 by improving metal uptake and tolerance. Front Microbiol.

[63] Gallardo-Benavente C, Carrión O, Todd JD, Pieretti JC, Seabra AB, Durán N (2019). Biosynthesis of CdS quantum dots mediated by volatile sulfur compounds released by antarctic Pseudomonas fragi. Front Microbiol.

[64] Qi S, Yang S, Chen J, Niu T, Yang Y, Xin B (2019). High-yield extracellular biosynthesis of ZnS quantum dots through a unique molecular mediation mechanism by the peculiar extracellular proteins secreted by a mixed sulfate reducing bacteria. ACS Appl Mater Interfaces.

[65] Xu J, Hu R, Wang Q, Wang P, Bao H (2019). Extracellular biosynthesis of biocompatible CdSe quantum dots. IET Nanobiotechnol.

[66] Gholami Z, Dadmehr M, Babaeian Jelodar N, Hosseini M, Pakdin Parizi A (2020). One-pot biosynthesis of CdS quantum dots through in vitro regeneration of hairy roots of Rhaphanus sativus L. and their apoptosis effect on MCF-7 and AGS cancerous human cell lines. Mater Rese Express.

[67] Cao K, Chen MM, Chang FY, Cheng YY, Tian LJ, Li F (2020). The biosynthesis of cadmium selenide quantum dots by Rhodotorulamucilaginosa PA-1 for photocatalysis. BiochemEng J.

[68] Qi S, Miao Y, Chen J, Chu H, Tian B, Wu B (2021). Controlled biosynthesis of ZnCdS quantum dots with visible-light-driven photocatalytic hydrogen production activity. Nanomaterials (Basel).

[69] Mi C, Wang Y, Zhang J, Huang H, Xu L, Wang S (2011). Biosynthesis and characterization of CdS quantum dots in genetically engineered Escherichia coli. J Biotechnol.

[70] Stürzenbaum SR, Höckner M, Panneerselvam A, Levitt J, Bouillard JS, Taniguchi S (2013). Biosynthesis of luminescent quantum dots in an earthworm. Nat Nanotechnol.

[71] Borovaya MN, Naumenko AP, Matvieieva NA, Blume YB, Yemets AI (2014). Biosynthesis of luminescent CdS quantum dots using plant hairy root culture. Nanoscale Res Lett.

[72] Ouyang W, Sun J (2016). Biosynthesis of silver sulfide quantum dots in wheat endosperm cells. Mater Lett.

[73] Green M, Haigh SJ, Lewis EA, Sandiford L, Burkitt-Gray M, Fleck R (2016). The biosynthesis of infrared-emitting quantum dots in Allium fistulosum. Sci Rep.

[74] Öcal N, Ceylan A, Duman F (2020). Intracellular biosynthesis of PbS quantum dots using Pseudomonas aeruginosa ATCC 27853: evaluation of antibacterial effects and DNA cleavage activities. World J Microbiol Biotechnol.

[75] Öcal N, Ceylan A, Duman F. Eco-friendly intracellular biosynthesis of CdS quantum dots using Pseudomonas aeruginosa: evaluation of antimicrobial effects and DNA cleavage activities. Recent Pat Nanotechnol 2021. 10.2174/1872210515666210719122353. 34825647

[76] Aubert T, Golovatenko AA, Samoli M, Lermusiaux L, Zinn T, Abécassis B (2022). General expression for the size-dependent optical properties of quantum dots. Nano Lett.

[77] Borghei YS, Hosseinkhani S (2022). “Semiconductor quantum dots” in biomedical opportunities. J Lumin.

[78] Amraei S, Eslami G, Taherpour A, Hashemi A (2022). Relationship between MOX genes and antibiotic resistance in Klebsiella pneumoniae strains in nosocomial infections. Micro Nano Bio Asp.

[79] Amraei S, Eslami G, Taherpour A, Hashemi A (2022). The role of ACT and FOX genes in Klebsiella pneumoniae strains isolated from hospitalized patients. Micro Nano Bio Asp.

[80] Ojha AK, Rajasekaran R, Pandey AK, Dutta A, Seesala VS, Das SK, et al. Nanotheranostics: nanoparticles applications, perspectives, and challenges. In: Borse V, Chandra P, Srivastava R, eds. BioSensing, Theranostics, and Medical Devices. Singapore: Springer; 2022. p. 345-76. 10.1007/978-981-16-2782-8_14.

[81] Nadar SS, Patil SP, Kelkar RK, Patil NP, Pise PV, Tiwari MS, et al. Nanobiomaterials for bioimaging. In: Liu HH, Shokuhfar T, Ghosh S, eds. Nanotechnology in Medicine and Biology. Elsevier; 2022. p. 189-234. 10.1016/b978-0-12-819469-0.00001-0.

[82] Roy S, Bobde Y, Ghosh B, Chakraborty C (2021). Targeted bioimaging of cancer cells using free folic acid-sensitive molybdenum disulfide quantum dots through fluorescence “turn-off”. ACS Appl Bio Mater.

[83] Galiyeva P, Rinnert H, Bouguet-Bonnet S, Leclerc S, Balan L, Alem H (2021). Mn-doped quinary Ag-In-Ga-Zn-S quantum dots for dual-modal imaging. ACS Omega.

[84] Martins CSM, LaGrow AP, Prior JAV (2022). Quantum dots for cancer-related mirna monitoring. ACS Sensors.

[85] Ncapayi V, Ninan N, Lebepe TC, Parani S, Girija AR, Bright R (2021). Diagnosis of prostate cancer and prostatitis using near infra-red fluorescent AgInSe/ZnS quantum dots. Int J Mol Sci.

[86] Fatima I, Rahdar A, Sargazi S, Barani M, Hassanisaadi M, Thakur VK (2021). Quantum dots: synthesis, antibody conjugation, and HER2-receptor targeting for breast cancer therapy. J FunctBiomater.

[87] Kim J, Hwang DW, Jung HS, Kim KW, Pham XH, Lee SH (2022). High-quantum yield alloy-typed core/shell CdSeZnS/ZnS quantum dots for bio-applications. J Nanobiotechnology.

[88] Sun X, Shi M, Zhang C, Yuan J, Yin M, Du S (2021). Fluorescent Ag–In–S/ZnS quantum dots for tumor drainage lymph node imaging in vivo. ACS Appl Nano Mater.

[89] Ding S, Zhang N, Lyu Z, Zhu W, Chang YC, Hu X (2021). Protein-based nanomaterials and nanosystems for biomedical applications: a review. Mater Today.

[90] Alavi M, Hamidi M. Passive and active targeting in cancer therapy by liposomes and lipid nanoparticles. Drug Metab Pers Ther 2019;34(1). 10.1515/dmpt-2018-0032. 30707682

[91] Ding S, Khan AI, Cai X, Song Y, Lyu Z, Du D (2020). Overcoming blood–brain barrier transport: advances in nanoparticle-based drug delivery strategies. Mater Today.

[92] Alavi M, Martinez F, Delgado DR, Tinjacá DA (2022). Anticancer and antibacterial activities of embelin: micro and nano aspects. Micro Nano Bio Asp.

[93] Alavi M, Kowalski R, Capasso R, Coutinho HD, Rose Alencar de Menezes I (2022). Various novel strategies for functionalization of gold and silver nanoparticles to hinder drug-resistant bacteria and cancer cells. Micro Nano Bio Asp.

[94] Kenchegowda M, Rahamathulla M, Hani U, Begum MY, Guruswamy S, Osmani RAM (2021). Smart nanocarriers as an emerging platform for cancer therapy: a review. Molecules.

[95] Tran HV, Ngo NM, Medhi R, Srinoi P, Liu T, Rittikulsittichai S (2022). multifunctional iron oxide magnetic nanoparticles for biomedical applications: a review. Materials (Basel).

[96] Ghosh S, Jayaram P, Kabekkodu SP, Satyamoorthy K (2022). Targeted drug delivery in cervical cancer: current perspectives. Eur J Pharmacol.

[97] Tandale P, Choudhary N, Singh J, Sharma A, Shukla A, Sriram P (2021). Fluorescent quantum dots: an insight on synthesis and potential biological application as drug carrier in cancer. BiochemBiophys Rep.

[98] Kabay G, DeCastro J, Altay A, Smith K, Lu HW, Capossela AM (2022). Emerging biosensing technologies for the diagnostics of viral infectious diseases. Adv Mater.

[99] Mohammadi MR, Omidi AH, Sabati H (2022). Current trends and new methods of detection of SARS-CoV-2 infection. Cell Mol Biomed Rep.

[100] Nasrin F, Chowdhury AD, Takemura K, Kozaki I, Honda H, Adegoke O (2020). Fluorometric virus detection platform using quantum dots-gold nanocomposites optimizing the linker length variation. Anal Chim Acta.

[101] Wang ZG, Liu SL, Pang DW (2021). Quantum dots: a promising fluorescent label for probing virus trafficking. Acc Chem Res.

[102] WHO. Who Coronavirus (COVID-19) Dashboard. 2022. Available from: https://covid19.who.int/.

[103] Li C, Zou Z, Liu H, Jin Y, Li G, Yuan C (2021). Synthesis of polystyrene-based fluorescent quantum dots nanolabel and its performance in H5N1 virus and SARS-CoV-2 antibody sensing. Talanta.

[104] Li Y, Ma P, Tao Q, Krause HJ, Yang S, Ding G (2021). Magnetic graphene quantum dots facilitate closed-tube one-step detection of SARS-CoV-2 with ultra-low field NMR relaxometry. Sens Actuators B Chem.

[105] Rabiee N, Ahmadi S, Jamalipour Soufi G, Hekmatnia A, Khatami M, Fatahi Y (2022). Quantum dots against SARS-CoV-2: diagnostic and therapeutic potentials. J Chem Technol Biotechnol.

[106] Zhao Y, Chen J, Hu Z, Chen Y, Tao Y, Wang L (2022). All-solid-state SARS-CoV-2 protein biosensor employing colloidal quantum dots-modified electrode. BiosensBioelectron.

[107] Chen R, Kan L, Duan F, He L, Wang M, Cui J (2021). Surface plasmon resonance aptasensor based on niobium carbide MXene quantum dots for nucleocapsid of SARS-CoV-2 detection. Mikrochim Acta.

[108] Jia J, Ao L, Luo Y, Liao T, Huang L, Zhuo D (2022). Quantum dots assembly enhanced and dual-antigen sandwich structured lateral flow immunoassay of SARS-CoV-2 antibody with simultaneously high sensitivity and specificity. BiosensBioelectron.

[109] Rezanejade Bardajee G, Zamani M, Sharifi M (2021). Efficient and versatile application of fluorescence DNA-conjugated CdTe quantum dots nanoprobe for detection of a specific target DNA of SARS Cov-2 virus. Langmuir.

[110] Gorshkov K, Susumu K, Wolak M, Oh E (2022). Fluorescent quantum dots enable SARS-CoV-2 antiviral drug discovery and development. Expert Opin Drug Discov.

